# Antibiotic administration *exacerbates* acute graft vs. host disease-induced bone marrow and spleen damage in lymphopenic mice

**DOI:** 10.1371/journal.pone.0254845

**Published:** 2021-08-06

**Authors:** Brianyell McDaniel Mims, Josue Enriquez, Andrea Pires dos Santos, Yava Jones-Hall, Scot Dowd, Kathryn L. Furr, Matthew B. Grisham

**Affiliations:** 1 Department of Immunology and Molecular Microbiology, Texas Tech University Health Sciences Center, Lubbock, TX, United States of America; 2 College of Veterinary Medicine, Department of Comparative Pathobiology, Purdue University, West Lafayette, IN, United States of America; 3 College of Veterinary Medicine and Biomedical Sciences, Department of Veterinary Pathobiology, Texas A&M University, College Station, TX, United States of America; 4 MR DNA (Molecular Research), Shallowater, TX, United States of America; Laikon Hospital, GREECE

## Abstract

**Background:**

Hematopoietic stem cell transplantation is a potential cure for certain life-threatening malignant and nonmalignant diseases. However, experimental and clinical studies have demonstrated that pre-transplant myeloablative conditioning damages the gut leading to translocation of intestinal bacteria and the development of acute graft vs. host disease (aGVHD). The overall objective of this study was to determine whether administration of broad spectrum antibiotics (Abx) affects the onset and/or severity of aGVHD in lymphopenic mice that were *not* subjected to toxic, pre-transplant conditioning.

**Results:**

We found that treatment of NK cell-depleted recombination activating gene-1-deficient (-NK/RAG) recipients with an Abx cocktail containing vancomycin and neomycin for 7 days prior to and 4 weeks following adoptive transfer of allogeneic CD4^+^ T cells, exacerbated the development of aGVHD-induced BM failure and spleen damage when compared to untreated–NK/RAG recipients engrafted with syngeneic or allogeneic T cells. Abx-treated mice exhibited severe anemia and monocytopenia as well as marked reductions in BM- and spleen-residing immune cells. Blinded histopathological analysis confirmed that Abx-treated mice engrafted with allogeneic T cells suffered significantly more damage to the BM and spleen than did untreated mice engrafted with allogeneic T cells. Abx-induced exacerbation of BM and spleen damage correlated with a dramatic reduction in fecal bacterial diversity, marked loss of anaerobic bacteria and remarkable expansion of potentially pathogenic bacteria.

**Conclusions:**

We conclude that continuous Abx treatment may aggravate aGVHD-induced tissue damage by reducing short chain fatty acid-producing anaerobes (e.g. *Clostridium*, *Blautia*) and/or by promoting the expansion of pathobionts (e.g. *Akkermansia*) and opportunistic pathogens (*Cronobacter)*.

## Introduction

Hematopoietic stem cell transplantation (HSCT) is a potential cure for different life-threatening malignant and nonmalignant diseases [1, 2]. Donor HSCs may be derived from bone marrow, peripheral blood, or umbilical cord blood. One of the most important factors that determines the outcome of HSCT is the identification of donors whose major histocompatibility complex I (MHC I) and MHC II are matched as closely as possible. Preclinical and clinical evidence demonstrate that engraftment of autologous HSCs (derived from recipient) or syngeneic HSCs (obtained from identical twin) produce better outcomes for patients by promoting robust engraftment, differentiation and expansion of healthy blood and immune cells. Unfortunately, the likelihood of obtaining siblings or other related family members who are fully or nearly MHC matched with the recipient is only 25% [3]. This reality has prompted clinicians to expand the pool of potential donors to include first degree relatives or unrelated donors who share one copy of chromosome 6 containing the MHC loci. These donors are referred to as *allogeneic* or *haploidentical* donors. By including these donors who are partially-mismatched with respect to recipient MHC, >95% of all patients who require HSCT are able to receive this treatment [3]. Although the use of allogeneic HSCT donors has greatly expanded the use of HSCT to treat refractory/recurring malignancies or blood disorders, ~50% of patients receiving this treatment will develop a potentially lethal, multi-organ inflammatory disease called acute graft vs. host disease (aGVHD) [1]. Clinically, aGVHD is characterized by inflammatory tissue damage in the gastrointestinal (GI) tract, skin, liver and lungs within the first 100 days following transplantation, [1, 4]. In addition, aGVHD may also damage the bone marrow (BM) and lymphoid tissue (spleen, thymus, lymph nodes) creating prolonged immunodeficiency that is characterized by pancytopenia, anemia and thrombocytopenia [5–9]. Development of aGVHD-induced BM failure and LT hypoplasia greatly increases the risk of infections and bleeding which accounts for ~30% of patient deaths with this disease [9]. Even if patients do survive this disease, they are at increased risk for developing diarrhea, nausea, vomiting, abdominal pain, oral ulcerations and dermal sclerosis. Although allogeneic HSCs are partially matched with respect to recipient MHC proteins and peptides, T cells residing within the HSC graft will recognize recipient MHC antigens as foreign (i.e. non-self) and launch a cascade of immunological responses that culminate in T cell-mediated inflammatory tissue damage to one or more tissues.

The protocol for performing HSCT requires that recipients be treated with some form of myeloablative therapy (e.g. lethal irradiation and/or cytotoxic chemotherapy) to destroy their bone marrow. This pre-transplant conditioning protocol creates immunological space that facilitates the engraftment, proliferation and differentiation of healthy HSCs into all of the cellular elements of blood. Unfortunately, myeloablative conditioning also damages the gut as well as other tissues. There is good evidence suggesting that injury to the intestinal tract plays an important role in the development of aGVHD by facilitating the translocation of intestinal bacteria and/or their components into gut tissue [1, 10–17]. These studies, coupled to the well-known clinical observation that patients undergoing allogeneic HSCT may develop severe neutropenia following myeloablative conditioning, have prompted investigators to assess the protective effects of antibiotic (Abx) administration prior to and/or following pre-transplant conditioning and HSC engraftment. Some studies have reported that Abx treatment of mice or humans undergoing HSCT attenuates the onset and/or severity of aGVHD [10, 18]. In contrast, other preclinical and clinical studies have demonstrated that administration of certain Abx may exacerbate aGVHD-induced intestinal damage and increase mortality by damaging bacterial communities that are critical for maintaining intestinal epithelial cell viability and mucosal barrier function [15, 18–20].

These observations prompted us to determine what effect, if any, continuous Abx administration has on the development of aGVHD in mice that are not subjected to myeloablation-induced intestinal injury. We hypothesized that in the absence of intestinal mucosal barrier disruption, commensal bacteria would play little or no role in the development of aGVHD. To test this hypothesis, we used our recently described mouse model of aGVHD that does not use toxic, pre-transplant conditioning [21]. We found that treatment of NK cell-depleted recombination activating gene-1-deficient (-NK/RAG1) recipients with an Abx cocktail containing vancomycin and neomycin for 7 days prior to and 4 weeks following adoptive transfer of allogeneic CD4^+^ T cells exacerbated aGVHD-induced bone marrow and spleen damage when compared to untreated mice engrafted with allogeneic or syngeneic T cells. The Abx-induced exacerbation of BM and spleen damage correlated with a dramatic reduction in fecal bacterial diversity, marked loss of anaerobic bacteria and remarkable expansion of potentially pathogenic bacteria. The mechanisms by which prolonged Abx administration amplifies allogeneic T cell-mediated tissue damage are discussed.

## Materials and methods

### Mice

Eight- to ten-week-old male wild-type (WT) C57BL/6 (Bl6) and C57Bl6/J RAG-1^-/-^ (RAG1^-/-^) mice and WT BALB/cJ (Balb/c) mice were purchased from Jackson Laboratory (Bar Harbor, ME) and maintained in the LARC facility at TTUHSC. All experimental procedures involving the use of animals were reviewed and approved by the Institutional Animal Care and Use Committee of TTUHSC and performed according to the criteria outlined by the National Institutes of Health. Mice were housed in ventilated micro-isolator cages at 22–24°C on 6:00am-6:00pm light cycle under specific pathogen free (SPF)/barrier conditions. Mice were screened semiannually for mouse hepatitis virus, mouse parvovirus (MPV1, MPV2, MPV3), minute virus of mice, mouse norovirus, Theiler’s murine encephalomyelitis virus, mouse rotavirus, Sendai virus, *Mycoplasma pulmonis*, pneumoniavirus, reovirus 3, lymphocytic choriomeningitis virus, Ectromelia virus and ectoparasites. All animals in our study were found to be free of the microorganisms mentioned above. The rooms and each individual cage were subjected to positive pressure relative to the outside environment to prevent microbial contamination. Cages were sterilized and furnished with wood chip bedding (7090 Sani-Chips, Harlan^®^ Laboratories Inc., Indianapolis, IN) and cotton material for nest construction. Animals were provided irradiated Prolab Isopro RMH 3000 (LabDiet, St. Louis, MO) rodent chow and tap water *ad libitum*. Mice were housed 3–4 per cage upon arrival from the vendor. The animals were then randomized into the different groups by first marking (via Sharpie pen) the tails of each mouse with one, two, three or no markings. Mice were then distributed into different cages such that each cage contained the four, differently marked mice. It is known that mice housed in the same cage will share their microbiota via coprophagy producing microbial communities that are similar but not identical to mice housed in other cages. In order to minimize these “cage effects”, we used a modification of the method of Rodriguez-Palacios et. al. [22]. Briefly, mice were removed from their cages each week at which time the soiled bedding from each cage was removed, combined and mixed and then distributed into clean cages. Different combinations of the 4 marked mice were added back to the cages ensuring that the same 4 mice were not housed together.

### Antibiotic (Abx) preparation

Based upon the work of Shen et. al. [23], we used a cocktail of two, non-absorbable Abx that we have previously demonstrated to be very effective in attenuating chronic colitis in mice [24]. Briefly, vancomycin (0.5 g/L; Sigma-Aldrich), neomycin (1.0 g/L; Sigma-Aldrich) and the artificial sweetener aspartame (Asp; 3.75 g/L; Sigma-Aldrich) were dissolved in tap water and administered *ad libitum*. Vancomycin is a glycopeptide that inhibits the 2^nd^ stage of cell wall synthesis of *gram-positive* bacteria whereas neomycin is an aminoglycoside that primarily targets *gram-negative* bacteria via its ability to inhibit protein synthesis through irreversible binding to the 30S ribosomal subunit.

### Abx treatment and induction of aGVHD

A total of 26 RAG1^-/-^ mice were randomized into 4 different groups in which mice were pretreated for 7 days with the artificial sweetener Asp alone or Asp plus the Abx cocktail in their drinking water using a minor modification of the method described by Shen et. al. [23, 24]. During the 7-day pretreatment, all mice were injected *(i*.*p)* with NK1.1 mAb (250 μg in 0.5ml PBS; clone PK136) at 48 and 24 hours prior to T cell transfer to deplete NK cells. We have found that this treatment protocol reduces CD335-expressing NK cells by ~90% when assessed at 24 hrs following the second injection (21). NK cell depleted RAG1^-/-^ (–NK/RAG) mice were then injected *(i*.*p*.*)* with 5 x10^6^ FACS-sorted allogeneic (Balb/c) or syngeneic (Bl6) CD4^+^CD25^-^ T cells. Mice were continuously maintained on drinking water containing Asp alone (-Abx) or Asp+Abx (+Abx) for 4 weeks following T cell injection producing the following 4 groups: -Abx/Syngeneic mice [8], +Abx/Syngeneic (N = 4), -Abx/Allogeneic (N = 8) and +Abx/Allogeneic (N = 6) mice. Mice who lost ≥20% of their original body weight were designated as moribund and were euthanized.

### Tissue preparation for blinded histological evaluation

Prior to euthanasia, aliquots of whole blood were obtained from anesthetized mice for hematocrit determination and plasma preparation. Following euthanasia, lungs were infused with a warm (37^○^C) 1% agarose solution into the trachea using a syringe with a 21G needle to inflate the lungs. Following euthanasia samples of skin, ears, lung, liver, spleen and colon from each animal were excised, fixed in 10% neutral phosphate-buffered (PBS) formalin and stored at 25°C. The tissue was then embedded in paraffin, sectioned (5 μm) and stained with hematoxylin and eosin. For bone and bone marrow preparation, femurs and humerus from euthanized mice were excised and all soft tissue (e.g. skin and muscle) was removed. Remaining bone was fixed in IBF Fixative containing isopropanol, barium chloride and formalin. These tissues were then decalcified using Mild HCl Decalcifier solution, embedded in paraffin, sectioned (5 μm) and stained with hematoxylin and eosin.

### Blinded histological evaluation

Representative sections of colon, lung, liver, spleen and skin were scored in a blinded fashion by our collaborator Dr. Yava Jones-Hall as previously described [21]. Briefly, the severity and distribution of inflammation in the colonic mucosa was quantified as mild to severe using a score of 0–3. Leukocyte distribution was identified as being present only in the lamina propria (1), extending to the submucosa (2) or extending to the serosa (3). Necrosis was assessed as mild, moderate, or severe with scores of 1–3, respectively. Goblet cell loss was denoted as being focal, multifocal, or diffuse with scores of 1–3, respectively. The number of crypt abscesses was quantified per 10 high power fields and quantified as 1–2, 3–4 and greater than 4 present (scores of 1–3, respectively). A score of 0 was assigned for each criterion not noted. Total disease score ranges from 0 to a maximum of 18 points based upon summation of each assigned criterion. Representative sections of the skin and ears were also assessed in a blinded fashion in which the pathology in the dermis and epidermis were evaluated using a minor modification of the established scoring system by Kaplan et. al. [25]. Epidermal damage was assessed as mild, moderate or severe (scores 1–3, respectively). Dermal collagen content, an indicator of dermal fibrosis, was assessed as slightly altered with a mild increase, moderate increase, or marked increase (scores 1–3, respectively). Inflammation was assessed as focal, multifocal or widespread (scores 1–3, respectively). A score of 0 was assigned for each criterion not noted. Total disease score ranged from 0 to a maximum of 9 points based upon summation of each assigned criterion. Blinded histopathological analysis of liver samples included the disease score assessed by the number of bile ducts involved and the severity of ductal inflammation and injury as described by Kaplan et. al. [25]. This was quantified 1–4 on the basis of few tracts having mild involvement; numerous tracts involved, but only mild disease; majority of tracts involved and having moderate disease; most tracts involved with severe disease. Bile duct injury was manifested by bile duct hyperplasia, periportal fibrosis, nuclear hyperchromasia, nuclear crowding, infiltrating lymphocytes, and cytoplasmic vacuolation and was assessed as mild, moderate or severe (scores 1–3, respectively). Inflammation analysis of periportal, centrilobular and midzonal regions were combined. In these regions, infiltration of leukocytes was assessed as mild, moderate or severe (scores 1–3, respectively). A score of 0 was assigned for each criterion not noted. Total disease score ranged from 0 to a maximum of 10 points based upon summation of each assigned criterion. Representative H&E stained sections of the lung from each mouse were evaluated as described above.

The histopathologic diagnoses criteria in the lungs that are described in the NIH consensus development project on criteria for clinical trials in chronic GVHD were used with minor modifications to assess inflammation and fibrosis in the lungs of these mice [26]. Inflammation was characterized as (1) lymphocytic bronchiolitis (LB) without subepithelial fibrosis affecting <25% of section; (2) LB without subepithelial fibrosis affecting 25–50% of section; (3) LB with subepithelial fibrosis affecting>50% of section. Fibrosis was semi-quantified as (1) mild in most bronchioles or (2) constrictive bronchiolitis obliterans (CBO) present. An additional point was given if mucostasis or aggregates of foamy macrophages were present or there was bronchiectasis present. A score of 0 was assigned for each criterion not represented in the section or present to a minimal/insignificant degree. Total disease score ranged from 0 to a maximum of 7 points based upon summation of each assigned criterion. Both spleens and bone marrow were scored in a blinded fashion by our collaborator Dr. Andrea Pires dos Santos as previously described [21]. Briefly, spleen scores were based on different aspects of the lesions including the presence of the red and white pulp, T zone lymphocytes, and presence of follicles (scores 0, present in the right amount; 1, mild decrease; 2, moderate decrease; 3, marked decrease), as well as extramedullary hematopoiesis, mantle cell zone, and the red to white pulp ratio (scores 0, present in the right amount; 1, mild increase; 2, moderate increase; 3, marked increase) for a maximum organ score of 24. Femur and humerus scores were based upon the extent and severity of the lesion characterized by hypocellularity and ranged from 0-no abnormalities; 1-mild, proximal epiphysis; 2-moderate, epiphysis and proximal diaphysis; 3-marked, epiphysis and diaphysis; to 4-severe, bone marrow aplasia.

### Immune cell analyses

Numbers and phenotypes of each T cell population as well as numbers of CD11b^+^ myeloid and CD335^+^ NK cells in the spleen and bone marrow were quantified using flow cytometric analyses. Single cell suspensions were prepared from spleens as previously described [24]. Bone marrow cells were expelled from femurs and suspended in Tris-buffer with ammonium chloride to lyse red blood cells and then suspended in FACs buffer (1x PBS with 4% fetal calf serum). Splenocytes and bone marrow cells were stained with CD4 PE-Cy7, CD44 Horizon V450, CD25 PE, CD11b FITC, and CD335 AF647 antibodies and analyzed by flow cytometry.

### Plasma cytokine determinations

Plasma cytokine concentrations were quantified by flow cytometry using the Legendplex^TM^ multiplex bead assay that quantifies 13 different cytokines including TNF-α, IL-1β, IFN-γ, IL-6, IL-17A, IL-23, MCP-1, IL-12p70, IL-10, IL-27, IFN-β, and GM-CSF (BioLegend, Inc., San Diego, CA).

### Fecal bacterial density determinations

Fecal pellets were collected from mice in sterilized beakers immediately before treatment, prior to T cell transfer at 7 days of pretreatment and immediately before euthanasia. The pellets were stored at -80^○^C until processing. Microbial DNA was isolated from ~10 mg of fecal pellets from each mouse using Qiagen QiAmp power fecal DNA kit. Briefly, each sample was subjected to 2 rounds of bead beating for 60 seconds each at 4.0 m/s (resting one minute between rounds) and further processed using the manufacturer’s guidelines. Fecal DNA was stored at -80^○^C for downstream applications. Bacterial density was quantified using 16S rDNA bacterial primers, 5′-TCCTACGGGAGGCAGCAGT-3′ (forward primer) 5′-GGACTACCAGGGTATCTAATCCTGTT-3′ (reverse primer), and quantitative PCR using *E*. *coli* as a standard reference bacterium. *E*. *coli* were grown in LB broth until it reached an optical density of 1.25. The colony forming units (CFU) were determined by serial dilution of the bacteria and isolation of DNA. DNA standards were serially diluted 9 times which represented 16S rDNA gene copies ranging from 10^10^ to 10^2^. These DNA standards were plated alongside the DNA isolated from the fecal pellets that were collected from each mouse in 96 well plates for quantitative PCR using the primers and biorad SSO advanced supermix. The PCR program consisted of denaturation at 98^○^C for 3 min, denaturation at 98^○^C for 30 sec, and annealing at 60^○^C for 10 sec for 40 cycles. This data was then interpreted using Biorad Maestro software. This software interpolates unknown samples based on the standard plot and Ct values. Final DNA copy numbers were normalized to represent gene copies per ng of DNA.

### Bacterial 16S rDNA sequencing and bioinformatics analyses

The 16s rRNA v4 hypervariable region was targeted for amplification using the 515F (5’-GTGYCAGCMGCCGCGGTAA-3’) / 806R (5’-GGACTACNVGGGTWTCTAAT-3’) primer pair under the following thermal cycler conditions: 95°C for 5 minutes, followed by 30 cycles of 95°C for 30 seconds, 53°C for 40 seconds and 72°C for 1 minute, and a final elongation step at 72°C for 10 minutes. Following targeted 16s rDNA PCR, a limited-cycle PCR (10-cycle) amplification was utilized to bind Unique Dual-Indices (UDI) to the 16s PCR product for downstream multiplex sequencing. PCR products were checked on a 2% agarose gel to determine amplification success, including the relative intensity of bands. Samples were pooled together in equal proportions based on their molecular weight and DNA concentrations. Pooled samples were purified using calibrated SPRI beads. Next the pooled and purified PCR products were normalized to 4nM and prepared for sequencing on the Illumina NovaSeq 6000 following the manufacturer’s guidelines. Sequence data was processed using MR DNA analysis pipeline (MR DNA, Shallowater, TX, USA). In summary, paired-end sequences were joined, forward/reverse primer sequences removed, and all sequences < 150bp were filtered out. Sequences were then quality-filtered using a maximum expected error threshold of 1.0 and dereplicated. The dereplicated or unique sequences were denoised; unique sequences identified with sequencing and/or PCR point errors removed, followed by chimera removal, thereby providing a unique denoised sequence or zOTU. Final zOTUs were taxonomically classified using BLASTn against a curated database derived from NCBI (www.ncbi.nlm.nih.gov).

Microbiome analysis, including alpha- and beta-diversity, was performed using the Qiime2 analysis pipeline. The input for Qiime2 analysis was the raw sequencing data, which was downloaded from Illumina BaseSpace. After removing the forward/reverse primer sequences from the raw sequencing data using the MR DNA FASTq processor, sequences were imported into Qiime2. Quality control and sequence clustering were completed using DADA2, followed by phylogenetic tree construction using the SATé-enabled phylogenetic placement (SEPP) technique. Standard alpha- and beta-diversity metrics were included in the microbiome analyses, including observed OTUs, Shannon’s diversity index, and both unweighted and weighted UniFrac distance matrices. Taxonomic analysis was performed using a Naive Bayes classifier trained on the greengenes 13_8 97% OTUs. Based upon the relative abundance of specific phyla, genera and species identified by the MR DNA pipeline, statistical comparisons were made using ANOVA and Tukey’s post hoc multiple comparison test.

### Statistical analyses

All statistical analyses were performed using GraphPad Prism software. Significant differences between 2 groups were determined using Student’s T test. Significant differences among 3 or more groups were determined using one-way ANOVA and Tukey’s multiple comparison post hoc test.

## Results

### Adoptive transfer of allogeneic CD4^+^ T cells into antibiotic-treated recipients alters the cellular composition in blood, bone marrow and spleen

**Fig 1:**
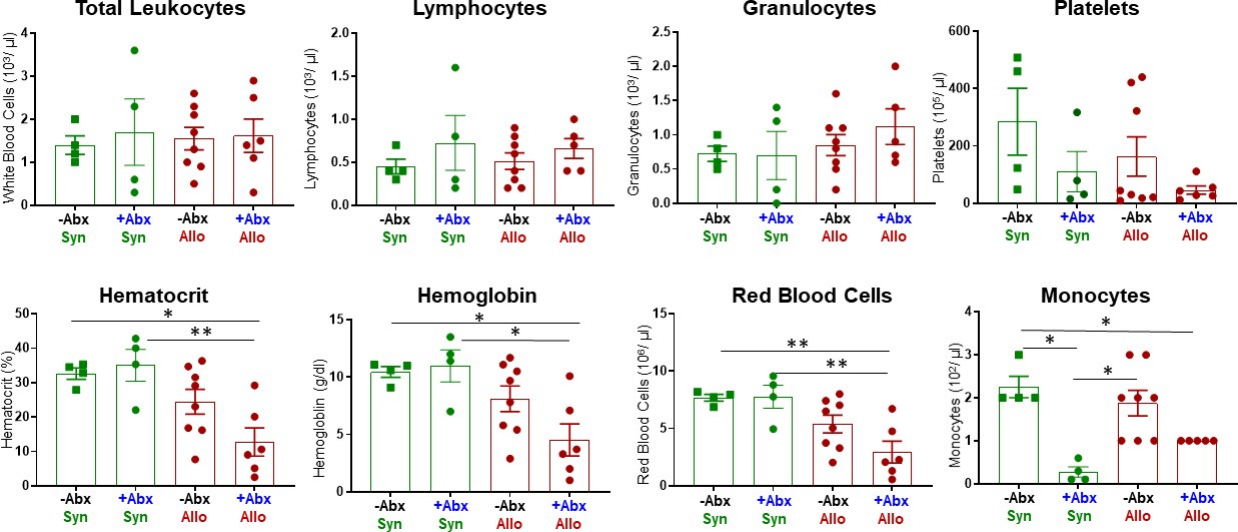
Complete blood cell counts (CBC). CBC analysis was quantified from EDTA-treated whole blood obtained from each mouse in the following 4 groups at 4 weeks post T cell transfer. Mice in the–**Abx/Syn** and -**Abx/Allo** groups represent–NK/RAG mice engrafted with syngeneic or allogeneic CD4^+^ T cells and treated with aspartame alone whereas **+Abx/Syn** and **+Abx/Allo** mice represent–NK/RAG mice engrafted with syngeneic or allogeneic T cells and treated with aspartame plus antibiotics. Asterisks (*) designate significant differences between groups (*p < 0.05, **p <0.01).

**Fig 2:**
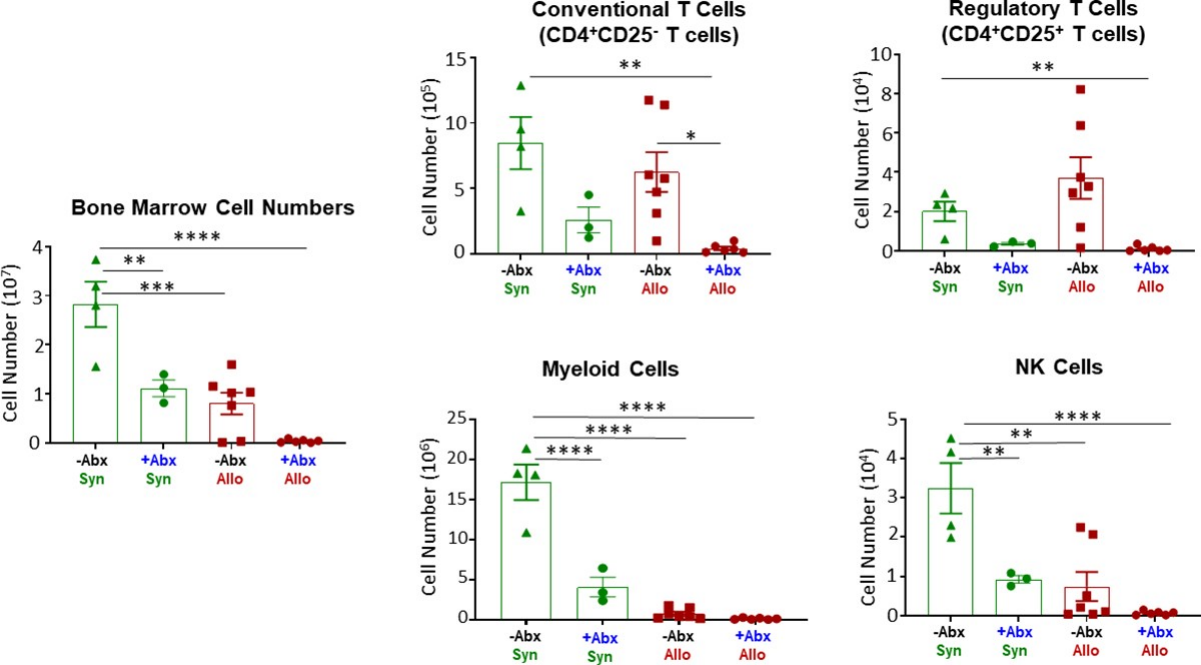
Acute GVHD-induced bone marrow damage. Total bone marrow (BM) cell numbers as well as BM-residing immune cells were quantified at 4 weeks post T cell transfer for mice in the 4 groups described in Fig 1. Conventional (CD4^+^CD25^-^) T cells, regulatory T cells (Tregs; CD4^+^CD25^+^), myeloid (CD11b^+^) cells and NK (CD335^+^) cells were quantified by flow cytometry. Asterisks (*) designate significant differences between groups. (*p < 0.05, **p < 0.01, ***p<0.001, ****p<0.0001).

**Fig 3:**
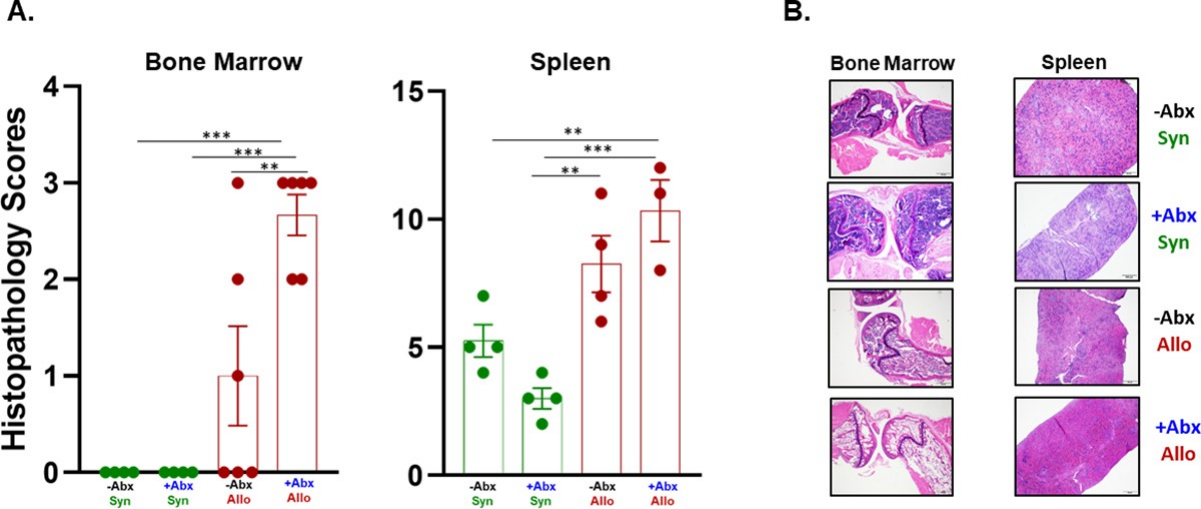
Histopathological analysis of bone marrow and spleen. **A).** Blinded histopathology scores were quantified for bone marrow and spleen obtained from mice in the 4 groups described in Fig 1 at 4 weeks post T cell transfer. **B).** Representative histological images of bone marrow and spleen from each group (40x total magnification). Asterisks (*) designate significant differences between groups. (**p <0.01; ***p<0.001).

It is well-known that bone marrow (BM) and lymphoid tissue (e.g. spleen, thymus) are particularly vulnerable to aGVHD-induced tissue damage [5–9]. We recently reported that engraftment of allogeneic T cells into -NK/RAG mice induced marked loss of BM and spleen cellularity that correlated with severe anemia and lymphopenia [21]. Therefore, we wished to determine the effects of antibiotic (Abx) treatment on the cellular composition of blood, BM and spleen. We found that treatment of–NK/RAG mice with Abx prior to and following adoptive transfer of allogeneic CD4^+^CD25^-^ T cells (+Abx/Allo mice) significantly reduced hematocrit, hemoglobin concentration and red blood cell numbers when compared to *untreated* recipients engrafted with syngeneic T cells (-Abx/Syn mice) (Fig 1). In addition, all three of these parameters were significantly reduced in +Abx/Allo when compared to Abx treated mice engrafted with syngeneic T cells (i.e. +Abx/Syn). In contrast, circulating number of monocytes in +Abx/Syn mice were significantly reduced when compared with the–Abx/Syn and -Abx/Allo mice (Fig 1). Blood levels of total leukocytes, lymphocytes, granulocytes and platelets were not different among the four groups (Fig 1). Because Abx treatment induced alterations in the numbers of certain blood cells, we next wished to determine whether Abx treatment altered the numbers and composition of cells residing within the BM and spleen. Similar to what we previously reported [21], adoptive transfer of allogeneic T cells into untreated–NK/RAG mice (-Abx/Allo) induced ~70% reduction in total BM cells when compared with–Abx/Syn mice (Fig 2). These large reductions in cell numbers were due primarily to the loss of myeloid (CD11b^+^) cells and NK (CD335^+^) cells (Fig 2). When allogeneic T cell-engrafted mice were treated with Abx (+Abx/Allo), we observed an even greater reduction of total BM cells when compared with–Abx/Syn mice that was associated with dramatic and significant reductions of conventional and regulatory T cells, myeloid cells and NK cells (Fig 2). In addition to these alterations in +Abx/Allo mice, we found that Abx-treated mice engrafted with syngeneic T cells (+Abx/Syn) reduced total BM cell numbers by ~60% that correlated with significant loss of myeloid cells and NK cells (Fig 2). Blinded histopathological analyses of femurs and humerus confirmed the dramatic loss of BM cellularity as indicated by the significant increases in histopathology scores in +Abx/Allo mice when compared with the other three groups (Fig 3A & 3B). Although we observed ~2.5-fold increase in the mean histopathology scores in the +Abx/Allo group when compared with the–Abx/Allo mice, this difference was not statistically significant (Fig 3A & 3B).

**Fig 4:**
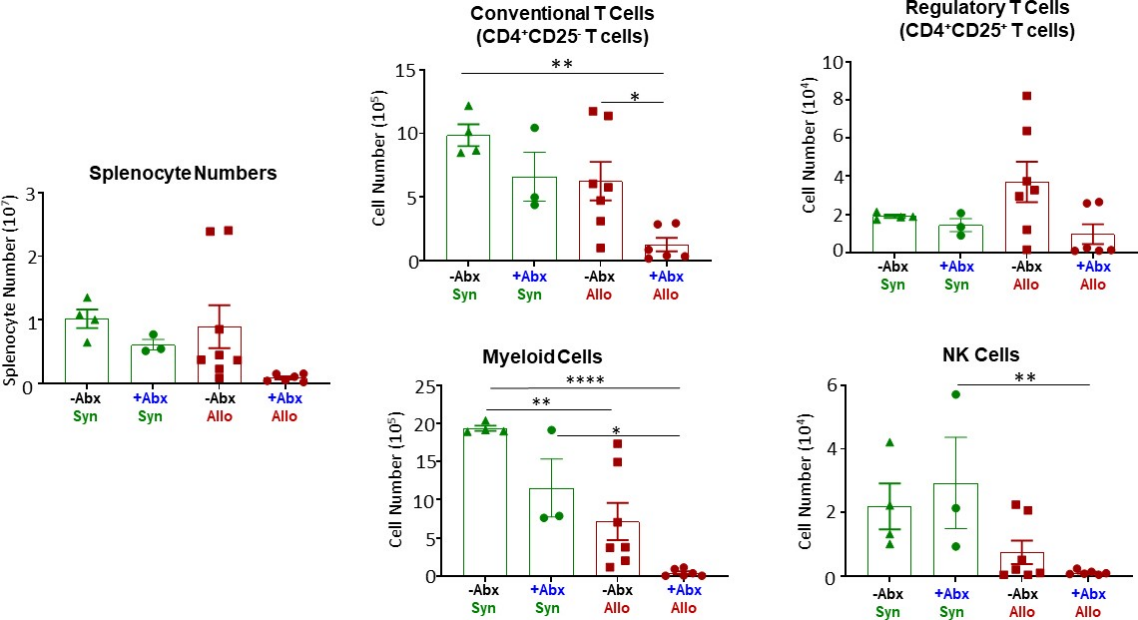
Acute GVHD-induced spleen hypoplasia. Spleen weights, splenocyte numbers and spleen-residing immune cells were quantified at 4 weeks post T cell transfer for mice in the 4 groups described in Fig 1. Conventional (CD4^+^CD25^-^) T cells, regulatory T cells (Tregs; CD4^+^CD25^+^), myeloid (CD11b^+^) cells and NK (CD335^+^) cells were quantified by flow cytometry. Asterisks (*) designate significant differences between groups. (*p < 0.05, **p < 0.01, ****p<0.0001).

Another tissue that is known to be a target for T cell-mediated damage during experimental and human aGVHD is the spleen [21, 27]. We found that total splenocyte numbers were reduced in +Abx/Allo mice when compared with the other three groups; however, this decrease was not statistically significant (Fig 4). Quantification of the different immune cells within the spleens of +Abx/Allo mice revealed large and significant reductions in the numbers of conventional T cells and myeloid cells when compared with–Abx/Syn mice (Fig 4). In addition, we found that the numbers of conventional T cells and myeloid cells were reduced further in the +Abx/Allo mice when compared with their–Abx/Allo counterparts (Fig 4). Interestingly, the total numbers of splenocytes, T cells, myeloid cells and/or NK cells in +Abx/Syn mice were not significantly reduced when compared to their–Abx/Syn controls (Fig 4). Blinded histopathological analyses of the spleens confirmed more extensive tissue disruption and reduced cellularity in +Abx/Allo mice when compared to the other three groups. We observed a significant 2-fold increase in histopathology scores for +Abx/Allo vs. -Abx/Syn mice and a significant 1.3-fold increase when compared to the -Abx/Allo group (Fig 3A & 3B). The increased histopathological scores in +Abx/Allo mice were due primarily to marked reductions of red and white pulp and reduced cellularity. When taken together, these data suggest that Abx treatment exacerbates aGVHD-induced BM and spleen damage.

We next wished to determine the effect adoptive transfer of allogeneic T cells have on the production of different inflammatory cytokines in the absence or presence of Abx. Surprisingly, we found that plasma levels of GM-CSF, IFN-β, IL-17A and IL-27 in–Abx/Allo mice were significantly reduced when compared to their–Abx/Syn controls(Table 1). Although we observed trends for increased concentrations of IFN-γ, MCP-1 and IL-6, they were not significant. We next wished to determine how Abx treatment affects cytokine concentration in–NK/RAG engrafted with syngeneic T cells. Although we did observe modest but significant increases in plasma concentrations of GM-CSF and IL-27 in +Abx/Syn mice, we also observed significant reductions in the levels of IFN-β, IL-17A and IL-27 in these same mice vs the–Abx/Syn group (Table 1). When we made a similar comparison between the +Abx/Allo and -Abx/Allo groups, we failed to observe significant differences in any of the 13 cytokines between the two groups. Finally, we found that +Abx/Allo mice had significant reduced plasma concentrations of GM-CSF, IL-23 and IL-27 when compared to +Abx/Syn mice (Table 1). Taken together, these data suggest that engraftment of allogeneic T cells into Abx-treated recipients enhances dramatically the production of IL-6 (Table 1). When taken together, these data suggest that Abx treatment of mice engrafted with syngeneic T cells is associated with both increased as well as decreased levels of certain cytokines when compared to their–Abx/Syn counterparts. In addition, these data suggest that engraftment of lymphopenic recipients with allogeneic T cells is associated with an overall reduction of several different inflammatory cytokines in the absence or presence of Abx.

**Table 1:** Plasma cytokine concentrations.

Cytokine	-ABX Syngeneic (A)	+ABX Syngeneic (B)	-ABX Allogeneic (C)	+ABX Allogeneic (D)	A vs B	A vs C	A vs D	B vs C	B vs D	C vs D
**GM-CSF**	11 ± 1.2515	15 ± 0.9224	1.6 ± 0.05	2.2 ± 0.7117	**0.0183**	**<0.0001**	**<0.0001**	**<0.0001**	**<0.0001**	0.9456
**IFN-β**	45 ± 6.2521	15 ± 3.9244	19 ± 0	19 ± 0	**0.0005**	**0.0017**	**0.0017**	0.893	0.893	>0.9999
IFN-γ	55 ± 12.3012	557 ± 262.7692	704 ± 292.0663	25 ± 13.7228	0.3172	0.1445	0.9995	0.9506	0.2728	0.1213
IL-10	35 ± 9.7734	65 ± 12.6423	172 ± 86.1524	362 ± 333.1905	0.9993	0.9415	0.5552	0.971	0.628	0.8614
IL-12p70	4.4 ± 2.0369	16.2625 ± 7.80	2.0 ± 0	2.0 ± 0	0.991	>0.9999	>0.9999	0.9848	0.9848	>0.9999
**IL-17A**	37 ± 14.101	13 ± 4.8681	1.4 ± 0	1.4 ± 0	0.1692	**0.0259**	**0.026**	0.6893	0.6899	>0.9999
IL-1α	16 ± 5.1799	55 ± 24.2075	27 ± 11.5831	159 ± 152.838	0.9838	0.9997	0.5782	0.9936	0.7805	0.6348
IL-1β	16 ± 2.4801	34 ± 12.7812	4.6 ± 0.3718	47 ± 42.65	0.941	0.9817	0.7714	0.7871	0.9772	0.5593
**IL-23**	63 ± 16.0228	134 ± 39.917	3.4 ± 0	3.6 ± 0.1883	0.1418	0.2587	0.261	**0.0049**	**0.005**	>0.9999
**IL-27**	88 ± 25.7518	182 ± 24.3157	9.2 ± 0.005	9.2 ± 0	**0.012**	**0.0382**	**0.0382**	**<0.0001**	**<0.0001**	>0.9999
IL-6	32 ± 16.727	110 ± 85.1967	619 ± 553.0006	2427 ± 2278.301	>0.9999	0.984	0.4983	0.9895	0.5248	0.7023
MCP-1	31 ± 4.238	111 ± 44.9372	101± 55.1991	414 ± 302.6086	0.9831	0.9885	0.3455	>0.9999	0.5334	0.5079
TNF-α	151 ± 37.5539	234 ± 101.1169	58±20.09	115 ± 91.1868	0.8483	0.7913	0.9832	0.3478	0.6551	0.9402

Plasma cytokine concentrations were quantified by flow cytometry at 4 weeks following T cell transfer. Mean±SEM cytokine concentrations for each group are reported. p values are reported for each group comparison.

### Adoptive transfer of syngeneic or allogeneic CD4^+^ T cells induces tissue-specific inflammation

**Fig 5:**
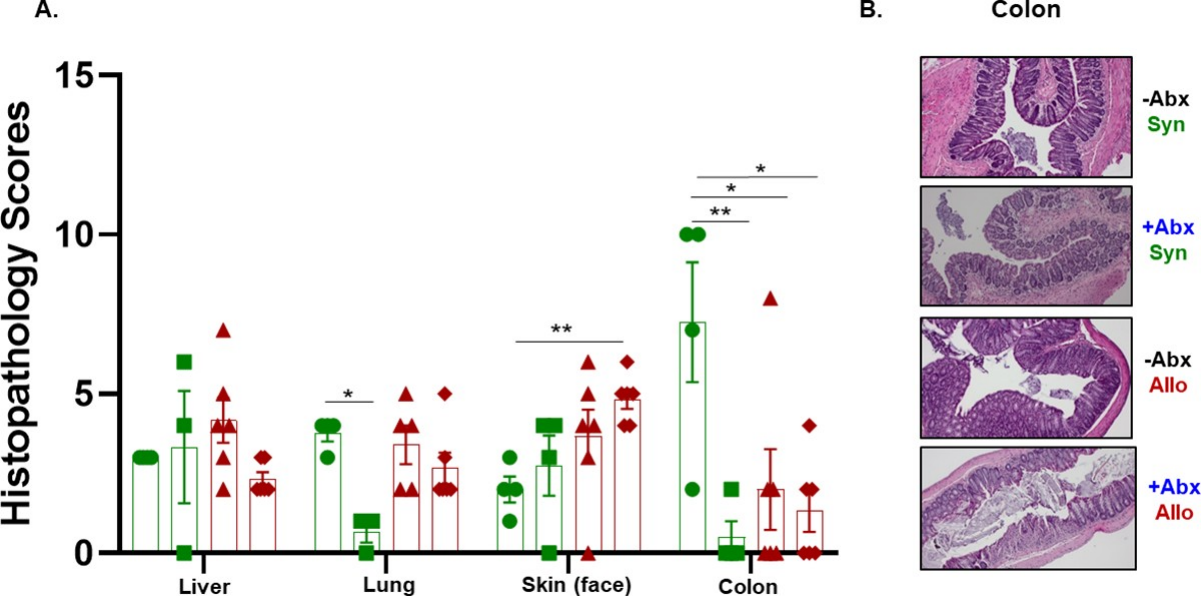
Histopathological analysis of different tissue. **A).** Blinded histopathology scores were quantified for the liver, lung, skin and colon obtained for mice in the 4 groups described in Fig 1 at 4 weeks post T cell transfer. Green circles and squares represent–Abx/Syn and +Abx/Syn whereas red triangles and diamonds represent–Abx/Allo and +Abx/Allo **B).** Representative histological images of the colon from each group (100x total magnification). Asterisks (*) designate significant differences between groups (*p < 0.05, **p < 0.01).

We recently reported that engraftment of allogeneic T cells into -NK/RAG mice induces liver and skin inflammation as well as BM and spleen damage [21]. Therefore, we wished to determine whether Abx treatment would affect the severity of inflammation in these and other tissues. Blinded histopathology analyses of the different tissues revealed no significant differences among the four groups with respect to liver inflammation; however, the histopathology scores for lungs in the +Abx/Syn mice was significantly less when compared to the–Abx/Syn and–Abx/Allo mice (Fig 5A). We also observed a significant increase in histopathology scores of the skin surrounding the eyes and nose of +Abx/Allo mice when compared to–Abx/Syn mice (Fig 5A). Surprisingly, and in contrast to our previous study [21], histopathological scores of the colon were dramatically and significantly increased in the–Abx/Syn group when compared to the other three groups (Fig 5A). We observed extensive colonic inflammation in the–Abx/Syn group that was characterized by bowel wall thickening with infiltration of large numbers of T cells and myeloid cells into the mucosa and submucosa as well as goblet cell loss and tissue necrosis (Fig 5A & 5B). Of note, Abx treatment of syngeneic T cell engrafted recipients (+Abx/Syn mice) significantly reduced colitis when compared to its–Abx/Syn counterpart (Fig 5A & 5B). Colitis was not observed in the–Abx/Allo and +Abx/Allo groups. Taken together, these data suggest that syngeneic T cells induce chronic colitis in the absence of Abx whereas engraftment of allogeneic T cells promotes skin inflammation, BM failure and spleen hypoplasia in the absence or presence of Abx. Table 2 summarizes the tissues with histopathological evidence of inflammatory tissue injury in each group.

**Table 2:** Summary of Inflammatory tissue injury in mice from each group.

Tissue Involved	-ABX Syngeneic	+ABX Syngeneic	-ABX Allogeneic	+ABX Allogeneic
Bone Marrow	**-**	**-**	**+**	**++**
Spleen	**-**	**-**	**+**	**++**
Liver	**-**	**-**	**-**	**-**
Lung	**-**	**-**	**-**	**-**
Skin	**-**	**-**	**+**	**+**
Colon	**++**	**-**	**-**	**-**

Inflammatory tissue damage in each tissue of the 4 groups is summarized based upon blinded histopathological analysis as described in the methods. Tissue with moderate (+), severe damage **(++)** or no discernable pathology **(-)** are noted.

### Alterations in bacterial density and composition in untreated or Abx-treated mice engrafted with T cells

**Fig 6:**
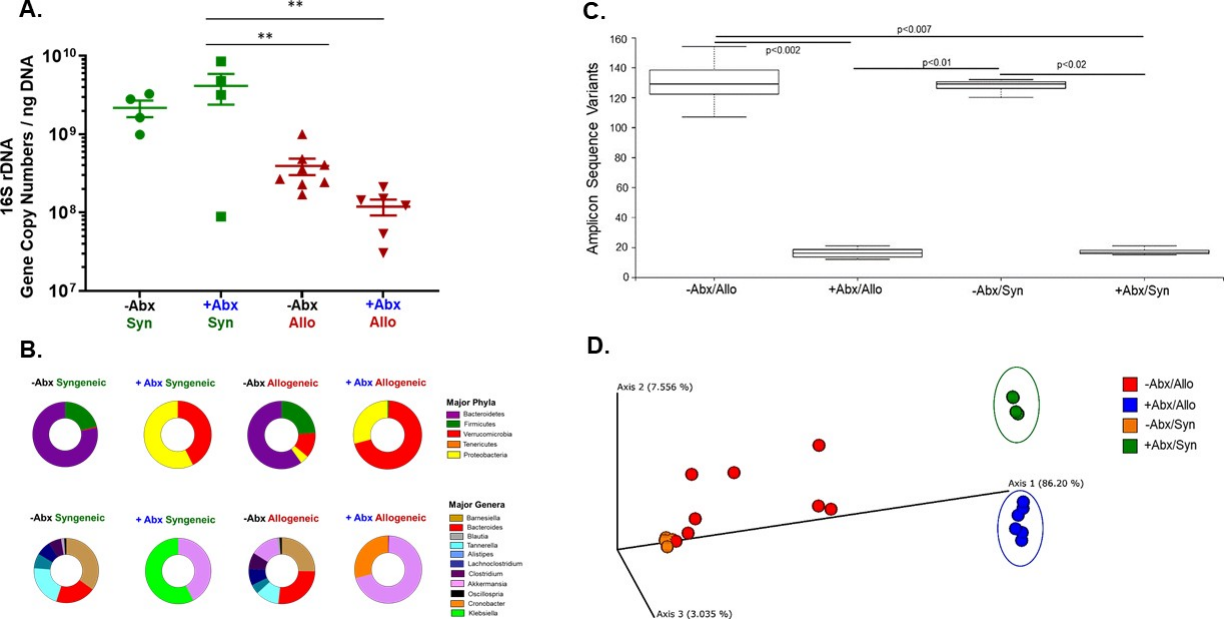
Alterations in fecal bacterial density and composition in untreated or Abx-treated mice. **A).** Fecal bacterial density was quantified at 4 weeks post T cell transfer. Data are expressed as 16s rRNA gene copy numbers per ng of DNA. Asterisks (*) designate significant differences among the 3 groups (**p< 0.01). **B).** Relative abundance of the major phyla and genera were determined using 16S rDNA amplicon sequencing of fecal DNA. **C).** Amplicon Sequence Variants (ASVs) with Kruskal-Wallis pairwise comparisons was used to quantify α diversity within each group. ASVs analysis revealed that the microbial diversity within the **+Abx/Syn** and **+Abx/Allo** groups were significantly reduced when compared with the–**Abx/Syn** and–**Abx/Allo** groups. **D)**. Principal Coordinate Analysis (PCoA) of weighted UniFrac distance was used to quantify differences between microbial communities (β Diversity). Using pairwise analysis of similarities, this plot reveals that the **+Abx/Syn** and **+Abx/Allo** groups were significantly different from each other as well as significantly different from the other two groups (p<0.05).

Previous work from our laboratory demonstrated, using a mouse model of inflammatory bowel disease (IBD), that continuous treatment of mice with a combination of neomycin and vancomycin in the drinking water (*ad libitum*) reduces fecal bacterial density and attenuated the development of chronic colitis suggesting that intestinal bacteria play an important role in the development of chronic gut inflammation [24]. Using this same treatment protocol, we quantified and compared the effects of a 7 day pretreatment of–NK/RAG mice with Asp alone (-Abx) or Asp plus Abx (+Abx) prior to and 4 weeks following T cell transfer on fecal bacterial density and composition. Surprisingly, we found that fecal bacterial density in the +Abx/Syn mice was not different from that observed for–Abx/Syn animals at 4 weeks post T cell transfer (Fig 6A). This was unexpected since bacterial density was reduced by several orders of magnitude following our 7 day pretreatment with Abx prior to T cell transfer (data not shown). Although we did observe a significant, 18-fold reduction in bacterial density in the +Abx/Allo group when compared with the +Abx/Syn group, it was not significantly different from its–Abx/Allo counterparts (Fig 6A). These data suggest that engraftment of allogeneic T cells reduces fecal bacterial density in the absence or presence of Abx when compared to +Abx/Syn mice. Another objective of this study was to determine how engraftment of syngeneic or allogeneic T cells into–NK/RAG mice in the absence or presence of Abx treatment alters the fecal bacterial composition. We found that adoptive transfer of *allogeneic* T cells into untreated recipients (-Abx/Allo) resulted in significant decreases in the relative abundance of the phyla Bacteriodetes and Spirochetes when compared with–Abx/Syn mice (Fig 6B and Table 3). We also noted significant decreases in the abundance of the genera *Tannerella and Enterococcus* in the–Abx/Allo group when compared with the–Abx/Syn group (Fig 6B and Table 3). These data suggested that engraftment of allogeneic T cells in untreated recipients altered the composition of the intestinal microbiota. Not surprisingly, when–NK/RAG mice were engrafted with syngeneic or allogeneic T cells and continuously treated with Abx, their fecal bacterial composition was dramatically altered when compared with their corresponding–Abx/Syn or -Abx/Allo groups. For example, we observed remarkable reductions in the relative abundance of Bacteroidetes and Firmicutes with corresponding increases in abundance of Verrucomicrobia and Proteobacteria in +Abx/Syn mice (Fig 6B and Table 3). These Abx-induced alterations were associated with large and significant decreases in the abundance of the anaerobic genera *Alistipes*, *Barnesiella*, *Blautia*, *Clostridium Lachnoclostrium and Tannerella* as well as large and significant increases in the relative abundance of *Akkermansia (42%)* and *Klebsiella (53%)* accounting for *~*95% of all genera identified in this group when compared to their–Abx/Syn counterparts (Fig 6B and Table 3). In addition, these Abx-induced alterations were associated with large and significant increases in the relative abundance of *Akkermansia muciniphila (43%) and Klebsiella oxytoca (54%)* when compared to–Abx/syn mice (Table 4). Continuous Abx treatment of allogeneic T cell-engrafted recipients induced similar but *not* identical alterations in the bacterial communities. Similar to what we observed in the +Abx/Allo mice, we found that the relative abundance of the same phyla (Bacteroidetes and Firmicutes) and major anaerobic genera (*Alistipes*, *Barnesiella*, *Blautia*, *Clostridium*, *Lachnoclostrium and Tannerella)* were dramatically reduced when compared to their–Abx/Allo counterparts (Fig 6B and Table 3). In contrast to +Abx/Syn mice, *Cronobacter (29%)* and *Akkermansia (70%)* represented 99% of all major genera in +Abx/Allo mice (Fig 6B and Table 3). These Abx-induced alterations were associated with large and significant increases in the abundance of the opportunistic pathogens *Cronobacter turicensis*, *Cronobacter sakasaki and Akkermansia mucinophilia* when compared to their–Abx/Allo counterparts (Table 4). Amplicon Sequence Variants (ASVs) analysis was used to quantify α Diversity within each group. We found that the number of ASVs within the +Abx/Syn and +Abx/Allo groups were dramatically and significantly reduced when compared to the–Abx/Syn and–Abx/Allo groups demonstrating Abx treatment markedly reduces microbial diversity (Fig 6C). Differences in microbial community structure between groups (i.e. β Diversity) was quantified using Principal Coordinate Analysis (PCoA) which takes into consideration both ASV composition and their relative abundance within each community (weighted UniFrac). We found that +Abx/Syn and +Abx/Allo mice were significantly different from each other as well as significantly different from the other two groups (Fig 6D). When taken together, these data suggest that different populations of CD4^+^ T cells may differentially shape microbial communities during Abx treatment.

**Table 3:** Alterations in the major bacterial phyla and genera in untreated or Abx-treated mice engrafted with T cells.

Major Phyla	-Abx Syngeneic (A)	+Abx Syngeneic (B)	-Abx Allogeneic (C)	+Abx Allogeneic (D)	A vs B	A vs C	A vs D	B vs C	B vs D	C vs D
Actinobacteria	0.08 ± 0.0162	0 ± 0	0.09 ± 0.0332	0 ± 0	0.094	0.0841	0.3008	0.9768	0.8379	0.9492
**Firmicutes**	21 ± 2.2472	0.0006 ± 0.0007	24 ± 2.8781	0.64 ± 0.5527	**0.000**	0.787	**< 0.0001**	**< 0.0001**	0.998	**< 0.0001**
**Verrucomicrobia**	0.46 ± 0.2332	42 ± 1.4958	12 ± 3.9370	70 ± 2.1699	**< 0.0001**	0.102	**< 0.0001**	**< 0.0001**	**0.000**	**< 0.0001**
**Proteobacteria**	0.02 ± 0.0069	58 ± 1.4970	4 ± 2.4575	29 ± 2.1752	**< 0.0001**	0.610	**< 0.0001**	**< 0.0001**	**< 0.0001**	**< 0.0001**
**Cyanobacteria**	0.003 ± 0.0013	0.005 ± 0.0011	0.0004 ± 0.0004	0.004 ± 0.0014	0.743	0.193	0.949	**0.020**	0.938	0.034
**Bacteroidetes**	78 ± 2.0790	0.0013 ± 0.0008	60 ± 4.8684	0 ± 0	**< 0.0001**	0.013	**< 0.0001**	**< 0.0001**	1.000	**< 0.0001**
**Spirochaetes**	0.01 ± 0.0046	0 ± 0	0.004 ± 0.0015	0 ± 0	**0.001**	**0.007**	**0.001**	0.497	1.00	0.391
Tenericutes	0.006 ± 0.0016	0.008 ± 0.0030	0.04 ± 0.0195	0.002 ± 0.0010	0.3918	0.144	0.2454	0.9964	0.9998	0.9989
**Major Genera**	**-Abx Syngeneic (A)**	**+Abx Syngeneic (B)**	**-Abx Allogeneic (C)**	**+Abx Allogeneic (D)**	**A vs B**	**A vs C**	**A vs D**	**B vs C**	**B vs D**	**C vs D**
**Barnesiella**	33 ± 2.3391	0.0006 ± 0.0007	22 ± 4.3585	0 ± 0	**< 0.0001**	0.157	**< 0.0001**	**0.001**	1.000	**0.000**
**Bacteroides**	19 ± 1.8067	0.0007 ± 0.0007	23 ± 2.4227	0 ± 0	**< 0.0001**	0.533	**< 0.0001**	**< 0.0001**	1.000	**< 0.0001**
**Tannerella**	19 ± 1.5987	0 ± 0	10 ± 1.4664	0 ± 0	**< 0.0001**	**0.000**	**< 0.0001**	**0.000**	1.000	**< 0.0001**
Lactobacillus	2.2 ± 1.5088	0 ± 0	2.7 ± 0.9694	0 ± 0	0.1218	0.9755	0.1973	0.4136	>0.9999	0.4903
**Alistipes**	7.4 ± 0.7917	0 ± 0	4.2 ± 1.6498	0 ± 0	**0.013**	0.338	**0.006**	0.135	1.000	**0.077**
**Lachnoclostridium**	6.8 ± 0.8338	0.0007 ± 0.0007	6.7 ± 1.4251	0 ± 0	**0.008**	1.000	**0.004**	**0.003**	1.000	**0.001**
**Clostridium**	5.9 ± 0.6817	0 ± 0	7.2 ± 1.3119	0.42 ± 0.3396	**0.013**	0.84	**0.011**	**0.001**	0.993	**0.000**
**Akkermansia**	0.46 ± 0.2332	42 ± 1.4958	12 ± 3.9370	70 ± 2.1699	**< 0.0001**	0.102	**< 0.0001**	**< 0.0001**	**0.000**	**< 0.0001**
**Blautia**	0.93 ± 0.1296	0 ± 0	1.1 ± 0.2552	0 ± 0	**0.049**	0.926	**0.028**	**0.005**	1.000	**0.002**
**Oscillospira**	1.0 ± 0.1779	0 ± 0	1.1 ± 0.2209	0 ± 0	**0.015**	0.981	**0.008**	**0.002**	1.000	**0.001**
**Candidatus phytoplasma**	0.004 ± 0.0008	0.008 ± 0.0030	0.0004 ± 0.0004	0.002 ± 0.0010	0.6764	0.1887	**0.0032**	0.7214	**0.0368**	0.3245
**Halospirulina**	0.003 ± 0.0013	0.005 ± 0.0011	0.0003 ± 0.0004	0.004 ± 0.0014	**0.0342**	0.1931	**0.0204**	0.9487	0.9386	0.7431
**Klebsiella**	0.001 ± 0.0008	53 ± 2.3553	0.005 ± 0.0033	0.046 ± 0.0292	**< 0.0001**	1.000	1.000	**< 0.0001**	**< 0.0001**	1.000
**Cronobacter**	0 ± 0	0.001 ± 0.0008	0.006 ± 0.0057	29 ± 2.0968	1.000	1.000	**< 0.0001**	1.000	**< 0.0001**	**< 0.0001**

Relative abundance of the major phyla and genera were determined using 16S rDNA amplicon sequencing of fecal DNA in the 4 groups described in Fig 1. The mean±SEM values (% relative abundance) for each group are reported. p values are reported for each group comparison.

**Table 4:** Alterations in the major bacterial species in untreated or Abx-treated mice engrafted with T cells.

Major Species	-Abx Syngeneic (A)	+Abx Syngeneic (B)	-Abx Allogeneic (C)	+Abx Allogeneic (D)	A vs. B	A vs. C	A vs. D	B vs. C	B vs. D	C vs. D
Blautia ruminococcus gnavus	0.45 ± 0.104	0 ± 0	0.26 ± 0.0906	0 ± 0.1011	0.0655	0.8343	0.2076	0.1477	0.7292	0.4752
**Barnesiella spp.**	33 ± 2.3390	0.0007 ± 0.0007	13 ± 4.3585	0 ± 5.7231	**0.006**	0.4895	**0.0213**	**0.0391**	0.66	0.1694
**Alistipes massiliensis**	7.4 ± 0.7918	0 ± 0	2.1 ± 1.6498	0 ± 1.1369	**0.0295**	0.4484	**0.0596**	0.2174	0.8483	0.4739
**Cronobacter turicensis**	0 ± 0	0.0006 ± 0.0007	0.004 ± 0	16 ± 2.9259	>0.9999	>0.9999	**0.0037**	>0.9999	**0.0037**	**0.0005**
**Cronobacter sakazakii**	0 ± 0	0.0006 ± 0.0006	0.0006 ± 0	10 ± 1.8832	>0.9999	>0.9999	**0.005**	>0.9999	**0.005**	**0.0007**
**Akkermansia muciniphila**	0.46 ± 0.2332	42 ± 1.4959	11 ± 3.9371	70 ± 11.4675	**0.039**	0.7963	**0.0023**	0.0989	0.8431	**0.0035**
Lachnoclostridium clostridium lavalense	0.068 ± 0.0191	0 ± 0	0.92 ± 0.4867	0 ± 0.0079	0.9994	0.3296	0.9995	0.2699	>0.9999	0.1383
Cronobacter muytjensii	0 ± 0	0 ± 0	0.006 ± 0.0054	2.7 ± 1.0417	>0.9999	>0.9999	0.2669	>0.9999	0.2669	0.1318
**Bacteroides acidifaciens**	19 ± 1.8049	0.0007 ± 0.0007	17 ± 2.4174	0 ± 2.9329	**0.0026**	0.7801	0.0075	**<0.0001**	0.6927	**<0.001**
Pantoea agglomerans	0 ± 0	3.8 ± 0.0007	3.1 ± 2.0973	0.001 ± 0.0009	>0.9999	0.4881	>0.9999	0.4883	>0.9999	0.3171
**Tannerella spp.**	19 ± 1.5987	0 ± 0	7.1 ± 1.4664	0 ± 3.2886	**0.0012**	0.0929	**0.005**	0.0634	0.5646	0.3628
**Klebsiella oxytoca**	0.001 ± 0.0008	53 ± 2.3550	0.006 ± 0.0033	0.0004 ± 4.3747	**<0.0001**	>0.9999	>0.9999	**<0.0001**	**<0.0001**	>0.9999

Relative abundance of the major species was determined using 16S rDNA amplicon sequencing of fecal DNA in the 4 groups described in Fig 1. The mean±SEM values (% relative abundance) for each group are reported. p values are reported for each group comparison.

## Discussion

This study was designed to examine the effects of Abx treatment on the development of aGVHD in mice that are *not* subjected to toxic, pre-transplant conditioning. We found that treatment of lymphopenic mice with a broad spectrum Abx cocktail prior to and following adoptive transfer of *allogeneic* T cells exacerbated T cell-mediated damage of the BM and spleen rendering these mice anemic and monocytopenic. These Abx-induced effects correlated with a large and significant reduction in fecal bacterial diversity, loss of short chain fatty acid (SCFA)-producing anaerobic bacteria and a marked expansion of potentially pathogenic bacteria. In addition to confirming our previous findings demonstrating that allogeneic CD4^+^ T cells are both necessary and sufficient to induce aGVHD in multiple tissues [21], the current studies revealed a few unexpected differences. For example, we found that engraftment of untreated–NK/RAG recipients with syngeneic T cells (–Abx/Syn mice) induced chronic colitis when compared to the other three groups (Fig 5A & 5B). Over the past few years, we have used engraftment of syngeneic T cells as a control group in this model of aGVHD since these T cells are incapable of inducing aGVHD in any tissue including the colon [21]. Although w and others have shown that adoptive transfer of naïve syngeneic CD4^+^ T cells into untreated RAG1^-/-^ recipients are capable of inducing chronic colitis, the onset and severity of disease is critically dependent upon the composition of intestinal bacteria [24, 28–30]. Indeed, our laboratory used this model of inflammatory bowel disease (IBD) for more than 16 years at our previous institution (LSU Health Sciences Center; LSUHSC) where we observed an incidence of moderate-to-severe colitis of ~90% [28]. Following relocation to our current institution, we noted a remarkable loss of disease phenotype that could be reestablished by colonizing RAG1^-/-^ recipients with fecal microbiota obtained from mice at LSUHSC [24]. It is well-known that this and several other mouse models of chronic gut inflammation are highly sensitive to and dependent upon the composition of the intestinal microbiota [24] and references therein]. Although we do not have a definitive explanation for why–NK/RAG mice engrafted with syngeneic CD4^+^ T cells developed chronic and unrelenting colitis in the current study, a likely explanation may be that administration of the artificial sweetener Asp alters colonic bacterial populations in such a way as to facilitate the development of colonic inflammation. Consistent with a role for intestinal bacteria in induction of chronic colitis, we found that +Abx/Syn mice did not develop disease (Fig 5).

It is well known that mice are averse to drinking water containing certain Abx thereby requiring the addition of an artificial sweetener (AS) to insure ingestion [31, 32]. Although addition of AS to the drinking water has proven useful for noninvasive dosing of rodents with different combinations of Abx, these sugar substitutes are known to alter intestinal bacterial communities [33, 34]. Rodriguez-Palacios and coworkers have demonstrated that treatment of mice with the AS *Splenda*, induced intestinal dysbiosis that was associated with expansion of Proteobacteria and infiltration of *E*. *coli* into the small intestinal interstitium [35]. Indeed, we found that administration of Asp to–NK/RAG mice prior to and following adoptive transfer of *syngeneic* T cells induced noticeable dysbiosis in these mice when compared to untreated mice engrafted with syngeneic T cells that did not develop chronic colitis. For example, we found that while continuous 4-week administration with Asp to mice engrafted with syngeneic T cells significantly reduced the relative abundance of Firmicutes and Verrucomicrobia as well as increased the abundance of Bacteriodetes when compared with mice that were engrafted with syngeneic T cells and given water alone *ad libitum* (S1 Table). These Asp-induced alterations were associated with significant *decreases* in the relative abundance of the genera *Akkemansia and Allobaculum* as well as significant increases in the abundance of *Bacteroides* and *Tannerella* (S1 Table). In addition, we observed significant increases in the *Tannerella spp* (S2 Table). Although it is not clear which of the different alterations is/are responsible for the development of chronic colitis in–Abx/Syn mice, species within *Bacteroides* and *Tannerella* may be likely candidates. For example, it has been shown that certain *Bacteroides* species may induce or are associated with the development of chronic colitis in different mouse models of IBD [24, 36–38]. In addition, certain *Tannerella* species found in oral bacteria have been shown to gain access to the intestinal lumen where they may contribute to the development of chronic gut inflammation [39]. When taken together, the current study is largely consistent with previous reports from our laboratory as well as others that alterations in the intestinal microbiota may dramatically affect the onset and/or severity of chronic gut inflammation [24]. Indeed, we show that Abx treatment markedly prevents the development of chronic colitis in +Abx/Syn mice (Fig 5A & 5B). These new data have important translational implications to the pathogenesis of human IBD. Epidemiological data suggest that consumption of certain dietary additives such as AS may be associated with increased risk of IBD ([35] and references therein). In addition to AS, other dietary constituents may alter the gut microbiota in such a way as to drive intestinal inflammation. A study by Chassaing et. al. demonstrated that ingestion of certain food emulsifiers induces gut dysbiosis and promotes colonic inflammation in mice [40].

Another difference we observed in the current vs. our previous study using the same mouse model of aGVHD [21], was the lack of significant liver inflammation in–Abx/Allo mice (Fig 5A). The reasons for this difference are not completely understood but again, are most likely related to alterations in commensal bacterial communities induced by prolonged Asp administration. It may be that Asp-induced dysbiosis alters the activation, differentiation and/or trafficking of disease producing T cells such that little or no inflammation could be observed in the liver. In fact, we observed significant dysbiosis in fecal samples obtained from–NK/RAG mice that were treated with Asp alone prior to and following adoptive transfer of *allogeneic* T cells (–Abx/Allo group) when compared to untreated, allogeneic T cell-engrafted mice. We found that administration of Asp to mice engrafted with allogeneic T cells significantly reduced the relative abundance of Firmicutes and *Lactobacillus* as well as an increased abundance of *Bacteroides*, *Akkermansia* and *Cronobacter turicensis* when compared to untreated mice engrafted with allogeneic T cells (S3 and S4 Tables). At the present time, it is not apparent which, if any of these alterations is/are responsible for suppressing hepatic inflammation in–Abx/Allo mice. Data presented in the current study also demonstrates that while prolonged Abx treatment dramatically alters microbial composition, it has a more modest effect on bacterial density (Fig 6). Our data suggest that certain species within *Cronobacter (C*. *turicensis*, *C*. *sakasaki)*, *Klebsiella (K*. *oxytoca) and Akkermansia* (*A*. *muciniphila*) are capable of substantial expansion in the continuous presence of Abx. These observations are consistent with those of other investigators who have reported on potential Abx resistance of these bacteria [41–44]. An alternative explanation for the expansion of these pathogens/pathobionts during prolonged Abx administration is that these bacteria may be protected from the anti-microbial activity of Abx provided that they reside within the gut embedded in their protective biofilm [45].

One of the most dramatic features of our model of aGVHD is the development of BM failure and spleen hypoplasia/atrophy (Figs 2–4) [21]. In human aGVHD-induced BM and lymphoid tissue damage, patients may experience prolonged immunodeficiency that is characterized by pancytopenia, thrombocytopenia and anemia [5–9]. It is thought that aGVHD-induced impairment of hematopoiesis and LT function occurs *rapidly* following allogeneic HSCT and may be mediated by CD4^+^ T cells [5, 8, 9, 27]. T cell-mediated damage to these tissues is associated with increased risk of infections and bleeding which account for approximately 30% of patient deaths following allogeneic aGVHD [9, 14, 46]. Data obtained in the current study demonstrated that Abx treatment prior to and following allogeneic T cell transfer markedly reduced BM and spleen cellularity when compared to their -Abx/Syn and/or -Abx/Allo counterparts (Figs 2 and 4). Abx-induced exacerbation of BM and spleen damage was characterized by tissue disruption and hypocellularity that was associated with loss of one or more immune cell populations (Figs 2–4). We also noted that while circulating numbers of monocytes as well as BM-associated myeloid cells and NK cells were significantly reduced in +Abx/Syn mice, none of the other CBC parameters nor splenocyte immune cell numbers were significantly reduced in this group vs. their–Abx/Syn counterparts (Figs 1, 2 and 4). These data suggest that BM is more sensitive than spleen to the Abx-induced alterations. The mechanisms by which Abx treatment exacerbates BM and spleen damage in +Abx/Allo mice are not known at the present time. It is well-known that IFN-γ, TNF-α and IL-6 are mediators of HSC progenitor cell cytotoxicity in mouse and human aGVHD [27, 47–51]. Although we observed trends for increases in plasma concentrations of IFN-γ and IL-6 in–Abx/Allo vs. -Abx/Syn mice, these differences were not significant (Table 1). Surprisingly, we found that those mice with confirmed BM and spleen damage (i.e.–Abx/Allo and +Abx/Allo; Figs 2 and 3) displayed significant reductions in circulating levels of GM-CSF, IFN-β, IL-17A and IL-27 (Table 1). Although these data would appear to exclude a role for these cytokines in allogeneic T cell mediated BM failure and spleen hypoplasia, it is quite possible that the generation of many of these inflammatory mediators (e.g. IFN-γ, TNF-α, IL-6, IL-17A, IL-23 etc.) may be greatly increased in the BM, spleen and skin at earlier time points during the activation, differentiation and expansion of allogeneic T cells. Current studies are underway to address this possibility.

Another potentially important mechanism by which prolonged Abx treatment exacerbates BM and/or spleen injury is by drastically reducing populations of intestinal bacteria whose cellular components and/or metabolites are critical for maintaining hematopoietic stem and/or progenitor cell (HSPCs) proliferation and differentiation [15]. Emerging data demonstrate that microbial-associated molecular patterns (MAMPS) play critical roles in regulating steady state hematopoiesis. Bone marrow mesenchymal stem cells (MSCs) express specific toll like receptors (TLRs) on their surface that bind extracellular MAMPS initiating signaling pathways that upregulate the expression of hematopoietic cytokines and growth factors [52, 53]. Iwamura et. al. reported that a peptidoglycan-derived component from E. coli called nucleotide binding oligomerization domain-containing protein 1 ligand (NOD1L), increases circulating levels of HSC proliferation-stimulating cytokines such as stem cell factor and thrombopoietin [54]. These growth factors are produced primarily by BM-residing MSCs and NOD1 *deficient* MSCs were *incapable* of producing stem cell factor and thrombopoietin [54]. Another recent study by Josefsdottir et. al. reported that a 2 week treatment of healthy wild type mice with a cocktail of four Abx, induced anemia and pancytopenia as well as depletion of BM HSCs and multipotent progenitor cells [55]. These investigators found that the effects of Abx treatment were duplicated in mice devoid of Stat-1 suggesting that loss of intestinal microbiota impairs MAMP-Stat1 signaling and disruption of progenitor cell maintenance and proliferation. Another recent study reported that interaction of MAMPs with MSC-associated TLRs initiates NOD1 signaling resulting in the production of type I interferon (IFN) [53]. The interaction of type I IFN with its receptor on the surface of HSCs and progenitor cells activates Stat1 signaling thereby inducing the expression of numerous mediators that promote hematopoiesis [53]. When taken together with these reports, our data suggest that certain commensal bacteria are crucial for the generation of erythrocytes and myeloid cells as well as T and B cells.

A third possible mechanism by which broad spectrum Abx treatment may aggravate allogeneic T cell-mediated BM and spleen damage is by reducing populations of the major SCFA-producing bacteria that include *Blautia*, *Barnesiella*, *Bacteroides and Clostridium* (Fig 6 and Table 3). Intraluminal production of acetate and butyrate by these anaerobic bacteria are known to be important for maintaining colonic epithelial cell health and mucosal barrier function [56, 57]. In particular, *Blautia* is known to be a major acetate and butyrate producer that represents one of the most abundant groups of bacteria within the human gastrointestinal tract comprising between 2.5% and 16% of the total microbiota [58]. Jenq et. al. recently reported that the presence of *Blautia* within the gut is associated with *reduced risk* of aGVHD in patients undergoing HSCT [59]. Thus, Abx-induced epithelial injury/dysfunction coupled to an increase in intestinal mucosal permeability would be expected to facilitate the translocation of luminal bacteria and their components into the tissue and systemic circulation where they could markedly affect HSPC function. It will be interesting to determine whether colonization of mice with different *Blautia* species prior to engraftment of allogeneic T cells will reduce the onset and/or severity of aGVHD-induced BM failure and spleen hypoplasia in our model. Currently, very little is known about the role of acetate and butyrate in hematopoiesis. Although Abx-mediated damage to the SCFA producing anaerobes is a reasonable explanation for BM and spleen damage, it must be noted that many of these same anaerobic bacteria are also markedly reduced in the +Abx/Syn group yet they exhibit substantially less BM and spleen hypoplasia (Fig 3). These data suggest that both allogeneic T cells and Abx treatment appear to be required for exacerbation of tissue damage.

It is also possible that prolonged Abx treatment may damage the BM and spleen by promoting the expansion of potentially pathogenic bacteria. We found that treatment of allogeneic T cell-engrafted mice with Abx remarkably increased the relative abundance of *Akkermansia and Cronobacter* when compared to–Abx/Allo mice (Fig 6 and Table 3). The only well-characterized specie within *Akkermansia* is the mucus-degrading bacterium *A*. *muciniphilia* (Table 4). Although *A*. *muciniphilia* is not normally considered a classic pathogen, it may be considered a pathobiont as its overabundance has been reported to be associated with severe experimental and clinical IBD [60–62]. Shono et. al. recently reported that treatment of mice or humans with a cocktail of broad spectrum Abx following allogeneic HSCT exacerbated aGVHD and increased mortality in both groups [19]. They found that Abx treatment was associated with colonic mucus degradation and impaired mucosal barrier function. These pathophysiological alterations to the gut were associated with an expansion of *A*. *muciniphilia* suggesting that loss of the protective mucus layer by these bacteria may represent a mechanism by which Abx-treatment promotes mucosal damage and translocation of intestinal bacteria into the systemic circulation [19]. In addition to *A*. *muciniphilia*, we observed a dramatic expansion of the species *C*. *turicensis and C*. *sakazakii* in Abx-treatment of mice engrafted with allogeneic T cells (Table 4). These gram negative, Enterobacteriaceae-associated species are known to be opportunistic, potentially Abx-resistant pathogens that infect children and adults [41, 42]. Alone or in the presence of large numbers of mucin-degrading *A*. *muciniphilia*, one or more of the *Cronobacter* species may gain access to the intestinal tissue and ultimately into the systemic circulation.

Deposition of certain gram negative bacterial products such as lipopolysaccharide (LPS) within the BM (and spleen) has been shown to mediate HSC dysfunction resulting in reduced output of immune cells. Takizawa et. al. demonstrated that prolonged adminstration of LPS or infection of mice with LPS-containing *Salmonella typhimurium* impaired HSC self-renewal and competitive repopulation activity [63]. These investigators showed that these detrimental effects were mediated by engagement of LPS with the pathogen sensing receptor, toll like receptor 4 (TLR4) expressed on the surface of HSCs. This interaction resulted in proliferative stress in BM-residing HSCs that culminated in reduced HSC output and hematopoietic repopulation [63]. In addition, Zhang and coworkers have reported that sepsis induced by LPS-containing *Pseudomonas aeruginosa* resulted in dysfunctional proliferation of HSPCs, depletion of myeloid progenitor cells and reduced output of myeloid cells [64]. The large and significant reductions in circulating monocytes as well as loss of CD11b^+^ myeloid cells in BM and spleen in our Abx-treated allogeneic T cell engrafted mice would be consistent with an LPS-mediated, TLR4-dependent block in myeloid cell differentiation as described above. Data generated in the current study are similar to those reported by Brown and coworker who found that prolonged Abx treatment of nonobese diabetic (NOD) newborns accelerated the development of diabetes that correlated with depletion of protective, SCFA-producing anaerobes and expansion of certain pathobionts (e.g. *A*. *muciniphilia*) [65]. In addition, our data appear to mirror those recently reported by Peled et. al who documented alterations in the fecal microbiota of 1,362 patients undergoing HSCT at four different centers in three different countries [66]. They observed a time-dependent disruption of fecal microbiota that was characterized by remarkable loss of fecal bacterial diversity with domination by specific pathogenic bacteria (e.g. *Enterococcus*, *Streptococcus*, *Klebsiella and/or Escherichia)* [66]. Because different pre-transplant conditioning protocols and Abx therapies varied across the different treatment centers in the US, Germany and Japan, it is difficult to determine the role that each of these variables plays in the development of intestinal dysbiosis [66]. The reasons why +Abx/Syn mice exibit less BM and spleen pathology despite a similar level of expansion of *A*. *muciniphilia* are not clear at the present time but may be due to a requirement for both allogeneic T cells and Abx as discussed above or to differences in the pathological potential of *K*. *oxytoca* (+Abx/Syn) *vs Cronobacter* species (+Abx/Allo) (Fig 6B and Table 4).

## Conclusion

The use of broad-spectrum Abx in patients undergoing gut-damaging myeloablative conditioning prior to allogeneic HSCT has been proposed to suppress the development of aGVHD and consequent life-threatening bacterial infections [10, 18]. In contrast, more recent reports have shown that administration of certain combinations of Abx may actually exacerbate aGVHD and increase mortality [15, 18–20]. Using our mouse model of aGVHD that does not require the use of injurious myeloablation, we found that treatment of lymphopenic mice with a broad spectrum Abx cocktail prior to and following adoptive transfer of allogeneic CD4^+^ T cells exacerbated the development of T cell-induced BM failure and spleen hypoplasia. These pathological conditions were associated with severe anemia and monocytopenia when compared to tissue damage in mice engrafted with allogeneic T cells that did not receive the Abx cocktail. Exacerbation of BM and spleen damage in mice treated with Abx correlated with a marked reduction in bacterial diversity, loss of anaerobic bacteria and notable expansion of potentially pathogenic bacteria. We conclude that Abx treatment may aggravate aGVHD-induced tissue damage by destroying protective, SCFA-producing bacteria (e.g. *Blautia*, *Clostridium etc*.) and/or by promoting the expansion of pathobionts (e.g., *Akkermansia*) and opportunistic pathogens (e.g. *Cronobacter)*.

## Supporting information

S1 Table Alterations in the major bacterial phyla and genera in untreated or aspartame-treated mice engrafted with syngeneic T cells. (DOCX)

S2 Table Alterations in the major bacterial species from untreated or aspartame-treated mice engrafted with syngeneic T cells. (DOCX)

S3 Table Alterations in the major phyla and genera from untreated or aspartame-treated mice engrafted with allogeneic T cells. (DOCX)

S4 Table Alterations in the major species from untreated or aspartame-treated mice engrafted with allogeneic T cells. (DOCX)

S1 Data (XLSX)

S2 Data (XLSX)

S3 Data (DOCX)

S4 Data (TXT)
