# Supplementary material for: Antibiotic administration exacerbates acute graft vs. host disease-induced bone marrow and spleen damage in lymphopenic mice
Source: PLoS One. 2021 Aug 6;16(8):e0254845. doi: 10.1371/journal.pone.0254845 (PMC8346256; doi:10.1371/journal.pone.0254845)
Supplement: S1 Table — (DOCX) [file pone.0254845.s001.docx]

**S1 Table.** **Alterations in the major bacterial phyla and genera in untreated or aspartame-treated mice engrafted with syngeneic T cells.**

| **Major Phyla** | **Untreated Syngeneic** | **Aspartame-Treated Syngeneic** | **p value** |
| --- | --- | --- | --- |
| **Bacteroidetes** | 46 ± 6.2868 | 78 ± 2.0790 | **<0.0001** |
| **Firmicutes** | 33 ± 3.9211 | 21 ± 2.2472 | **0.0282** |
| **Verrucomicrobia** | 20 ± 9.7661 | 0.5 ± 0.2332 | **< 0.0001** |
| Actinobacteria | 1.2 ± 0.3199 | 0.1 ± 0.0162 | 0.9994 |
| **Major Genera** | **Untreated Syngeneic** | **Aspartame-Treated**  **Syngeneic** | **p value** |
| *Barnesiella* | 34 ± 8.9705 | 33 ± 2.3391 | >0.9999 |
| ***Akkermansia*** | 20 ± 9.7661 | 0.5 ± 0.2332 | **<0.0001** |
| *Lactobacillus* | 13 ± 7.6721 | 2.0 ± 1.5088 | >0.9999 |
| ***Allobaculum*** | 12 ± 5.6139 | 0 ± 0.0042 | **<0.0001** |
| ***Bacteroides*** | 6.8 ± 3.4801 | 19 ± 1.8067 | **<0.0001** |
| *Alistipes* | 3.2 ± 1.5365 | 7.4 ± 0.7917 | 0.6763 |
| *Lachnoclostridium* | 2.4 ± 1.1411 | 7.0 ± 0.8338 | 0.5211 |
| *Clostridium* | 2.1 ± 0.5797 | 6.0 ± 0.6817 | 0.8274 |
| ***Tannerella*** | 1.7 ± 0.7605 | 20 ± 1.5987 | **<0.0001** |
| *Oscillospira* | 0.3 ± 0.0436 | 1.0 ± 0.1779 | >0.9999 |

The relative abundance of the major phyla and genera were quantified from feces obtained from untreated –NK/RAG mice engrafted with syngeneic T cells (Untreated Syngeneic) or mice treated with aspartame for 7 days prior to and 4 weeks following engraftment with syngeneic T cells (Aspartame-Treated Syngeneic). The mean±SEM values (% relative abundance) are reported for each group. Significant differences between the two groups are noted by bolded p values.
