# Supplementary material for: Antibiotic administration exacerbates acute graft vs. host disease-induced bone marrow and spleen damage in lymphopenic mice
Source: PLoS One. 2021 Aug 6;16(8):e0254845. doi: 10.1371/journal.pone.0254845 (PMC8346256; doi:10.1371/journal.pone.0254845)
Supplement: S2 Table — (DOCX) [file pone.0254845.s002.docx]

**S2 Table. Alterations in the major bacterial species from untreated or aspartame-treated mice engrafted with syngeneic T cells.**

| **Major Species** | **Untreated**  **Syngeneic** | **Aspartame-treated**  **Syngeneic** | **p value** |
| --- | --- | --- | --- |
| *Blautia ruminococcus gnavus* | 0.10 ± 0.0459 | 0.45 ± 0.1049 | 0.466 |
| *Alistipes massiliensis* | 3.2 ± 1.5365 | 7.4 ± 0.7917 | 0.502 |
| *Roseburia faecis* | 0.01 ± 0.0070 | 0.05 ± 0.0248 | 0.757 |
| *Intestinimonas butyriciproducens* | 0.01 ± 0.0031 | 0.02 ± 0.0116 | 0.757 |
| *Clostridium sp.* | 1.1 ± 0.3129 | 4.2 ± 0.5609 | 0.132 |
| *Akkermansia muciniphila* | 20 ± 9.7661 | 0.46 ± 0.2332 | 0.502 |
| *Blautia sp.* | 0.04 ± 0.0287 | 0.12 ± 0.0699 | 0.757 |
| *Allobaculum sp* | 12 ± 5.6133 | 0 ± 0 | 0.502 |
| *Lachnoclostridium clostridium hathewayi* | 0.23 ± 0.1589 | 1.5 ± 0.2983 | 0.305 |
| *Lachnoclostridium clostridium saccharolyticum* | 0.24 ± 0.2243 | 3.6 ± 1.242096 | 0.526 |
| *Erysipelatoclostridium clostridium cocleatum* | 0.24 ± 0.1211 | 0.01 ± 0.0055 | 0.526 |
| *Clostridium spp.* | 0.61 ± 0.0706 | 1.5 ± 0.2178 | 0.273 |
| *Citrobacter spp.* | 0.18 ± 0.1564 | 0.02 ± 0.0071 | 0.757 |
| *Oscillospira spp.* | 0.18 ± 0.0436 | 1.0 ± 0.1779 | 0.207 |
| *Bacteroides acidifaciens* | 7 ± 3.4801 | 19 ± 1.8048 | 0.286 |
| ***Tannerella spp.*** | 1.7 ± 0.7605 | 20 ± 1.5987 | **0.006** |
| *Lactobacillus johnsonii* | 13 ± 7.4311 | 2.0 ± 1.4428 | 0.663 |

The relative abundance of the major species was quantified from feces obtained from untreated –NK/RAG mice engrafted with syngeneic T cells (Untreated Syngeneic) or mice treated with aspartame for 7 days prior to and 4 weeks following engraftment with syngeneic T cells (Aspartame-Treated Syngeneic). The mean±SEM values (% relative abundance) are reported for each group. Significant differences between the two groups are noted by bolded p values.
