# Supplementary material for: Antibiotic administration exacerbates acute graft vs. host disease-induced bone marrow and spleen damage in lymphopenic mice
Source: PLoS One. 2021 Aug 6;16(8):e0254845. doi: 10.1371/journal.pone.0254845 (PMC8346256; doi:10.1371/journal.pone.0254845)
Supplement: S3 Table — (DOCX) [file pone.0254845.s003.docx]

**S3 Table.** **Alterations in the major phyla and genera from untreated or aspartame-treated mice engrafted with allogeneic T cells.**

| **Major Phyla** | **Untreated Allogeneic** | **Aspartame-Treated Allogeneic** | **p value** |
| --- | --- | --- | --- |
| Bacteroidetes | 53 ± 7.6843 | 60 ± 4.8684 | 0.6090 |
| **Firmicutes** | 42 ± 5.7625 | 24 ± 2.8781 | **0.0014** |
| Verrucomicrobia | 5.2 ± 3.2503 | 12 ± 3.9370 | 0.6090 |
| Proteobacteria | 0.10 ± 0.0765 | 4.1 ± 2.4575 | 0.9070 |
| **Major Genera** | **Untreated Allogeneic** | **Aspartame-Treated**  **Allogeneic** | **p value** |
| *Barnesiella* | 26 ± 2.3623 | 22 ± 4.3585 | 0.1505 |
| ***Lactobacillus*** | 16 ± 5.6031 | 2.7 ± 0.9694 | **<0.0001** |
| ***Bacteroides*** | 13 ± 3.1351 | 23 ± 2.4227 | **<0.0001** |
| *Tannerella* | 7.6 ± 2.0836 | 10 ± 1.4664 | 0.9974 |
| *Lachnoclostridium* | 7.2 ± 1.7161 | 6.7 ± 1.4251 | >0.9999 |
| *Alistipes* | 6.2 ± 1.9349 | 4.2 ± 1.6498 | >0.9999 |
| ***Akkermansia*** | 5.23.2503 | 12 ± 3.9370 | **0.0004** |
| *Clostridium* | 4.5 ± 1.5784 | 7.2 ± 1.3119 | 0.9919 |
| *Robinsoniella* | 4.3 ± 3.9002 | 1.1 ± 0.2552 | 0.8862 |
| *Eubacterium* | 1.3 ± 0.812883 | 1.2 ± 0.227206 | >0.9999 |
| *Lactococcus* | 3.9 ± 3.870794 | 0.001 ± 0.00076 | 0.4472 |
| *Dorea* | 1.0 ± 4.358478 | 0.3 ± 0.111252 | >0.9999 |

The Relative abundance of the major phyla and genera was quantified from feces obtained from

untreated –NK/RAG mice engrafted with allogeneic T cells (Untreated Allogeneic) or mice treated with aspartame for 7 days prior to and 4 weeks following engraftment with allogeneic T cells (Aspartame-Treated Allogeneic). The mean±SEM values (% relative abundance) are reported for each group. Significant differences between the two groups are noted by bolded p values.
