# Supplementary material for: Antibiotic administration exacerbates acute graft vs. host disease-induced bone marrow and spleen damage in lymphopenic mice
Source: PLoS One. 2021 Aug 6;16(8):e0254845. doi: 10.1371/journal.pone.0254845 (PMC8346256; doi:10.1371/journal.pone.0254845)
Supplement: S4 Table — (DOCX) [file pone.0254845.s004.docx]

**S4 Table.** **Alterations in the major species from untreated or aspartame-treated mice engrafted with allogeneic T cells.**

| **Major Species** | **Untreated**  **Allogeneic** | **Aspartame-Treated Allogeneic** | **p value** |
| --- | --- | --- | --- |
| *Lactobacillus salivarius* | 0.24 ± 0.2427 | 0 ± 0 | 0.8096 |
| ***Cronobacter turicensis*** | 0 ± 0 | 0.004 ± 0.0006 | **0.0070** |
| *Akkermansia muciniphila* | 5.2 ± 3.2503 | 11 ± 4.6331 | 0.8096 |
| *Clostridium fusiformis* | 0.15 ± 0.0633 | 0.04 ± 0.0243 | 0.5791 |
| *Lactobacillus intestinalis* | 0.02 ± 0.0241 | 0.001 ± 0 | 0.8096 |
| *Bacteroides xylanisolvens* | 0.03 ± 0.0102 | 0 ± 0.0016 | 0.8096 |
| *Enterococcus villorum* | 0.04 ± 0.0284 | 0.21 ± 0.0656 | 0.5054 |
| *Pseudobutyrivibrio spp.* | 0.003 ± 0.0012 | 0.17 ± 0.0955 | 0.7943 |
| *Staphylococcus sciuri* | 0.02 ± 0.0101 | 0.10 ± 0.0561 | 0.8096 |
| *Clostridium sulfatireducens* | 0.23 ± 0.1368 | 1.8 ± 0.7393 | 0.6686 |
| *Bacteroides acidifaciens* | 13 ± 3.1811 | 17 ± 3.0522 | 0.8096 |
| *Lactobacillus johnsonii* | 14 ± 4.9903 | 1.2 ± 0.4009 | 0.1071 |

The relative abundance of the major species was quantified from feces obtained from untreated –NK/RAG mice engrafted with allogeneic T cells (Untreated Allogeneic) or mice treated with aspartame for 7 days prior to and 4 weeks following engraftment with allogeneic T cells (Aspartame-Treated Allogenic). The mean±SEM values (% relative abundance) are reported for each group. Significant differences between the two groups are noted by bolded p values.
