# Supplementary material for: Antibiotic administration exacerbates acute graft vs. host disease-induced bone marrow and spleen damage in lymphopenic mice
Source: PLoS One. 2021 Aug 6;16(8):e0254845. doi: 10.1371/journal.pone.0254845 (PMC8346256; doi:10.1371/journal.pone.0254845)
Supplement: S3 Data — (DOCX) [file pone.0254845.s007.docx]

**Alpha Diversity- Amplicon Sequence Variants**

F1 Group-F 107

F2 Group-F 114

F3 Group-F 131

F4 Group-F 127

F5 Group-F 125

F6 Group-F 136

F7 Group-F 145

F8 Group-F 154

G1 Group-G 15

G2 Group-G 21

G3 Group-G 13

G4 Group-G 17

G5 Group-G 12

G6 Group-G 19

H1 Group-H 130

H2 Group-H 132

H3 Group-H 128

H4 Group-H 120

I1 Group-I 15

I2 Group-I 21

I3 Group-I 17

I4 Group-I 16

F1-F8 are the mice in the **–ABX/Allogeneic group**

G1-G6 are the mice in the **+ABX/Allogeneic group**

H1-H4 are the mice in the **–ABX/Syngeneic group**

I1-I4 are the mice in the **+ABX/Syngeneic group**
